# Engaging the open science framework in quantifying and tracing scientists’ research credits

**DOI:** 10.3389/fnint.2022.1028986

**Published:** 2023-01-12

**Authors:** Zhiyi Chen, Xuerong Liu, Kuan Miao, Xingya Liao, Xiaoling Zhang, Zhengzhi Feng, Hu Chuan-Peng

**Affiliations:** ^1^Department of Medical Psychology, Army Medical University, Chongqing, China; ^2^Experimental Research Center for Medical and Psychological Science (ERC-MPS), Army Medical University, Chongqing, China; ^3^School of Psychology, Southwest University, Chongqing, China; ^4^School of Psychology, Nanjing Normal University, Nanjing, China

**Keywords:** authorship, contributionship, early career researchers, gender equality, open science

## Abstract

Preface illustration. The “first-last-author-credit” hierarchy has long been dominated in the scientific incentive system despite intensive calling for contribution-based credits (author contribution statement). In the scientific communities, senior researchers would still make a decision to recommend one’s promotion based on first and last positions in authorship rather than their contributions. Similarly, in the job market, institutions would acknowledge one’s credit by positions in authorship in a study for faculty recruitment, while overlooking the author contribution statement at the end of studies. Thus, the current authorship system has brought on the risks underlying authorship disputes and race/gender inequalities in credit allocation heavily, especially for early career researchers and female scientists. In addition, this is one of the major barriers to extend teamwork and academic collaboration. On the contrary, scrambling for first and last positions leads to prominent credit inflation—that is to be observed—the number of co-first and co-corresponding authors has been increasing dramatically. Thus, we shall propose a new contributionship to acknowledge the author’s credit for an open science and quantitative framework to tackle these issues. Credit: ZC and XRL. 
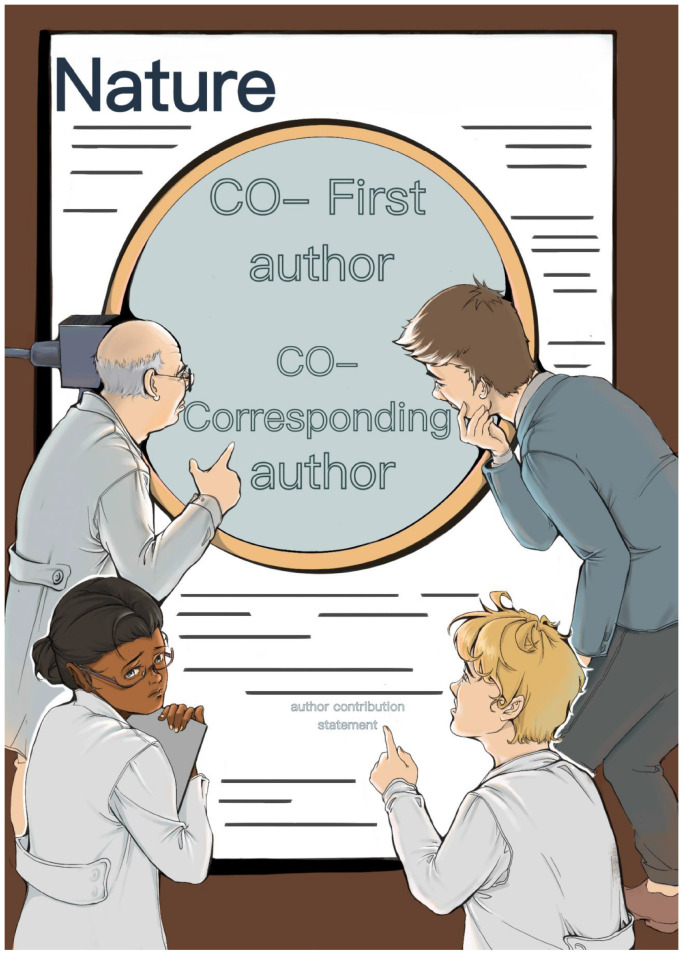

## Introduction

Debates on how to determine positions in research authorship have not subsided. An institution funded by the United States National Institute of Environmental Health Sciences (NIEHS) provided reports for investigating authorship disputes from 6,700 researchers in the world. It found out that nearly half of the respondents had suffered from naming disagreement, and 38% of them believed that they had experienced unfair authorship ranking (Smith et al., 2020). What makes this trend worrisome is the fact that the matter of authorship unfolds sharp gender inequalities in the scientific community, where female authors are arranged with more co-first (rather than the first) authors relative to what is applicable to male authors (Fleming, 2021). Furthermore, the scrambling for privileged positions in authorship arrangements is increasingly forcing early career researchers to distance themselves from scientific works and big-science collaborations, especially neuroscientists (Yager, 2007; Coles et al., 2022). Under the current scientific incentive system, it is an intuition that the credits a study can deliver for an author can be likened to the commercial values of a building, with the front location (position) indicating a high price (credit).

## Our efforts and challenges

It is no doubt that publishers have been aware of this circumstance and have made efforts to prioritize authors’ credits above authorship arrangement. Scientific journals have long been firm and enthusiastic in asking authors to state and clarify what scientific roles they played in the actualization of a research study. The confirmation and clarification are usually made in a purpose-built section entitled, “Author Contribution Statement (ACS)” (Vasilevsky et al., 2021). Fortunately, this statement has been increasingly accepted by mainstream publishers such as *Nature Springer*, *Elsevier*, and *Cell Press* for fostering contributionship. However, the absence of standard measurement for this claim of scientific roles in the ACS makes it rather difficult to specifically evaluate and determine an author’s deserved credits.

Furthermore, a machine-readable standardized statement called contributor roles taxonomy (CRediT) emerged to recognize scientific credits for authors based on the 15 categories, which have been broadly implemented thus far. That notwithstanding, another issue of concern in the matter of ACS is the fact that CRediT is not available for all the fields of scholarship such as literature and library science. Recently, the contribution role ontology (CRO), a system, developed by the National Center for Data to Health (NCDH) recommended that CRediT be extended to include more statements (Ross-Hellauer, 2022). The inclusion should add 50 categories for covering almost all the fields of scholarly interest—the roles of community, coordination, and so on.

Despite the huge progress made so far, the scientific community still has challenges and difficulties accepting contributionship as a better alternative. In addition, institutions and job markets disapprove of faculty recruitment or promotion that is based on the ACS. One major reason for this aversion is the apparent stiff competition for faculty positions requiring institutions to make decisions rapidly and directly for the numerous researchers who are in their early career phase. This makes it less likely to deliberately evaluate authors’ contributions claimed in studies one at a time. Moreover, another reason that could impede the spread of contributionship is the lack of quantifiable criteria for answering questions on how scientific credits that are based on authorship contributions could be evaluated. In addition, contributionship in the current tone is likely to expose an author’s credit to a high risk of inflation, which may provide an “infinite credit resource” in evaluating one’s contribution. The latter could make the scientific community fairly cautious in confronting such initiatives.

## Open science and quantitative framework

Open Science Framework (OSF) typically advocates three principles in knowledge production, which are transparency, equity, and accountability (Madhur and Avci, 2022). In practice, OSF recommends authors to pre-register their research proposal in the accessible repository beforehand, so as to enable detailed, original sampling methods, analytic plans, and hypotheses.

The OSF is a powerful vehicle for facilitating and driving transparency through a deliberate reduction in the manipulation of model parameters and results. Meanwhile, equity and accountability, the values that the OSF pursues, emphasize the equal right of all the authors fully involved in scientific work. However, the authors are required to shoulder the same accountability for the weights they contribute to the work.

Using the percentage-of-contribution indicator (PCI) and author CRediT score (ACS), we have proposed the quantitative framework as an alternative for acknowledging authors’ credits in authorship claims (Smith et al., 2018). Be that as it may, we reckon that two intrinsic pitfalls may occur in extending these quantitative systems. The first pitfall is the possibility of compounding impact factor (IF) in calculating authors’ contributions. Authorship has, so far, been broadly certified to recognize and make sure the author who makes more scientific contributions to research gets due credit for the study. This may, however, not be adequate for acknowledging contributions or values that are judged by a journal’s IF. Another flaw of the authorship system is the challenge of calculating the quantitative contributions of authors by absolute counts of categorical roles. In the process of estimating contribution—albeit it makes sense to consider the number of roles an author played in the CRediT category—it is usually unclear and difficult to determine the number of contributions that have been made for each role and how crucial each of the roles is for the study.

## Open science and quantitative contributionship

We propose a new framework that integrates Open Science principles and quantitative rules for acknowledging scientists’ credits in a study (see Box 1). More specifically, the framework recommends that authors pre-register and adopt the standardized authorship and contribution form (ACF) before the formal research procedure begins.^1^ The ACF requires authors to self-estimate their contributions quantitatively ranging from 0 to 1.0 (contribution coefficient) by referring to either the CRediT or CRO statement. It also requires authors to provide details illustrating what parts would be handled in the corresponding categorical roles. Moreover, traceable modifications toward contributionship could be allowed on the ground of authors’ consensus before pre-registering the ACF. In addition, the email addresses or any pathways accessible to each author should be given in the ACF. Once the work has been prepared for submission, the ACF should be designed to be in line with the pre-registration imperative in the online submission system. Any disparities compared with the pre-registration should be clearly stated in the ACS. Finally, the integrated ACF encompassing the authorship, contribution contexts/coefficients, and contacts would be generated automatically by the submission system and would be further printed at the head of the published study.

**BOX 1 Key steps for proposed contributionship framework.**

**Step 1: Pre-registration**

A standardized authorship and contribution form (ACF) requires the scientific communities to pre-register in repository or platform once research proposal is available:

**-Authorship:** *authors should be determined beforehand following the research proposal.*

**-Contribution:** *contributions should be described by contributor roles taxonomy (CRediT) or contribution role ontology (CRO) system in detail, including the illustration about which parts would be done in each category (e.g., drafting introduction at paragraph 1–3).*

**-Contribution coefficients:** *contribution coefficients should be provided grounded on actual contributions defined by CRediT or CRO system, but could be adjusted according to authors’ consensus; the sum of coefficients for all the authors is equal to one; individual coefficient should be marked alongside with author’s name.*

**-Contacts:** *authors should provide the accessible contact details alongside with contributions for the sake of facilitating correspondence and accountability.*

**Step 2: Formal submission**

Once the formal manuscript has been prepared, one author should be designated to submit it at online submission system:

**-Online submission system:** *author could upload the pre-registered ACF online submission system for automatic detection or fill ACF in system manually.*

**-Author contribution statement (ACS) conflicts:** *if any disparities emerged from the comparison to pre-registration, the reasons for modifying authors or contributions should be stated clearly in this section.*

**Step 3: Publication and after publication**

*Once manuscript has been published at a journal, this ACF would be generated automatically to attach at the HEAD of final manuscript for acknowledging credits for each author:*

**-Publication:** *ACF for final version should be attached at the first page of paper for detailing authorship/contributionship and history of modifying them; any disparities or modifications from pre-registration should be clearly claimed in ACF; individual coefficient should be marked alongside with author’s name.*

**-After publication:** *contributions and corresponding credits could be evaluated by ACF with specific contexts and coefficients for authors in job markets; each author should be accountable for giving response for concerns and queries that readers raise aiming at his/her contributions in the paper as reported in ACF.*

## Discussion

### Benefits and caveats

It is apparently rewarding to adopt the proposed authorship framework embracing the Open Science and quantitative contributionship systems. The framework could help drastically reduce authorship disputes by allocating credits to authors with the quantitative contribution method. The latter would be prominently beneficial for ensuring authoring equality in the increasing teamwork and academic collaboration system. The sequence determination authorship has been criticized for creating hurdles in academic collaborations as it assigns credits mostly by the first-and-last-author system (Hosseini, 2020). The more unfairly and unequally co-authors perceive they have been treated, the less motivation they have to concretize the teamwork they need. Thus, sharing credits with equal positions in this framework would be highly conducive to preventing scientific collaborations from suffering recurring authorship disputes.

**FIGURE 1:**
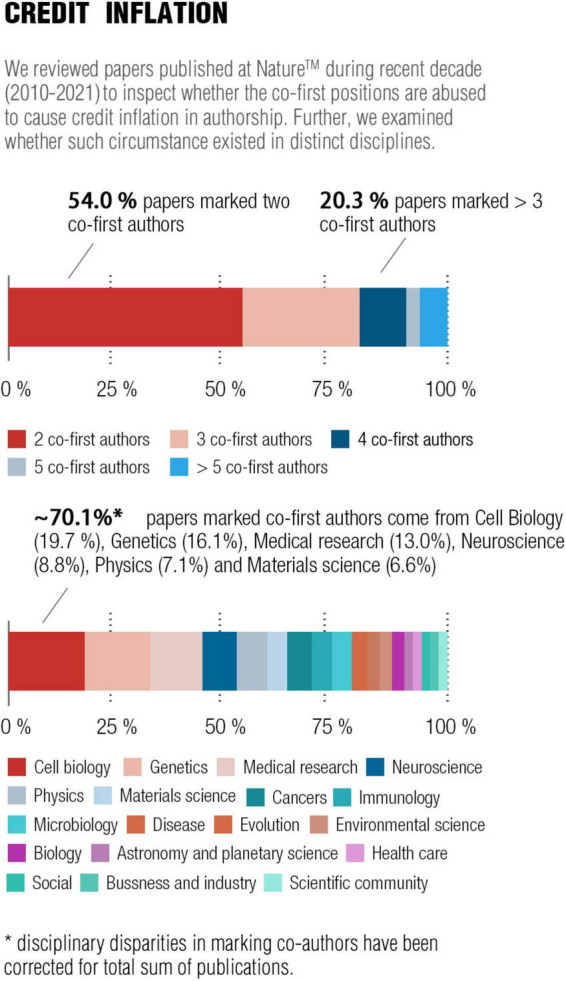
Credit inflation for co-first or co-corresponding positions in authorship. Data are acquired from Nature™ (https://www.nature.com) by reviewing authorship for all the research articles published in Nature during the recent decade.

Moreover, the tendencies for credit inflation may be controlled by quantitative contributionship. To tackle credit disputes in authorship, marking co-first and co-corresponding authors in one study is gradually becoming mainstream in the scientific community. Nevertheless, the trend appears to have been abused in the current form (Hornburg, 2018). We have reviewed studies published in Nature™ within this decade (2010–2021) in order to scrutinize this trend. Nearly half of the studies therein marked the co-first authors or co-corresponding authors, especially in the domains of cell biology, genetics, medial research, and neuroscience (see Figure 1). Thus, claiming “equally contributed” for the co-first or co-last positions may be an artifice for credits. This quantitative framework is required to clearly and transparently state authors’ contributions in authorship rather than some vague and unquantifiable mark, which facilitates the possible reduction of the guest-like co-first or co-corresponding authors. Furthermore, adding any additional co-authors would be at the expense of the decreasing average contribution coefficient, which propels the decision to mark co-authors prudently so as to limit credit inflation.

In addition, the transparency needed to determine authorship would be strengthened by the use of this framework. Pre-registration provides traceable and detectable access for observing the modification of authorship. It prevents honorary or guest authors from compromising the submission process. On the one hand, it seems to be a common configuration to add renowned researchers as honorary authors as a way of increasing the credits of the submitted articles and impressing the editors (Hornburg, 2018). On the other hand, the number of guest authors is sharply increasing in studies as an “interpersonal transaction” for extending the academic social network. Therefore, transparent reports on how authorship forms and the contributions of each author make this framework significant would be a promising way of cracking down on such naming misconducts.

It is worth highlighting that this framework makes accountability feasible for contributionship and, thus, maybe a blow to the widespread fake-paper factories and academic fraud systems. In Chinese Academy Science (CAS) institutions, the requisites for applying for tenured positions are at least eight studies that are published in high-profile journals (at least IF > 5.0) during a period of 3–6 years. The publications should have a varied outlook of independence and corresponding authorship. Even if doctors who are in their early career phase work in low-ranking institutions (e.g., level-2 local hospitals in China), their promotion will require at least three studies with authorship positions. Additional positions in authorship hardly have any credits in the current scientific incentive system, at least in mainland China. Consequently, the serious career pressure often mounted on researchers to publish studies as either first or corresponding authors is one of the main reasons why young researchers indulge in academic fraud at the expense of an auspicious long-term career prospect. They settle for predatory journals or fake publishing firms.

The new framework provides transparent ways for authors to obtain credits they merit based on accountable and traceable contributions by quantitative metrics. A key advantage of this framework is that it discourages academic misconduct from authors. On the contrary, transparently reporting specific contributions of authors with contact information makes accountability feasible. It is also beneficial for investigative agencies that might want to examine specific authors who may have been reported for misconduct.

The caveats for implementing this contribution framework should be reiterated. Unexpected academic bullying may occur when the contribution coefficients are rated. Even if the extreme battles or disputes for authorship positions are significantly eased, it is hard to estimate the risk of what—bullying—junior researchers could suffer in the coefficient competition. There is less power for them to go against the senior researchers in allocating the coefficients. Another challenge is the high risk of abuse as stated in “Goodhart’s Law” (Abualigah et al., 2021; Abualigah et al., 2022; Agushaka et al., 2022). Although each author receives credit fairly as expected, evaluating scientific credits for each of them may be abused when the coefficient is overly considered.

### A framework beyond initiative

This is not a conceptual call for acknowledging each author’s credits but aims to provide pragmatic ways to shift authorship from the “first-last-author-credit” hierarchy to a transparent, equal, and accountable contributionship. Compared to previous ones, this contributionship presents a quite mild and balanced scheme, neither in abolishing the “sequence-authorship-credit” hierarchy nor in overreaching absolutely objective index (Smith et al., 2018), which promises the acceleration to popularize this credit system.

Technically speaking, however, few hurdles would meet the implementation of this contributionship in the current technological framework. By mainstreaming the online submission system and ORCID with standardized machine-readable CRediT or CRO statements, all the processes that the contributionship requires could be easily supported. We have prepared a fictitious submission system for simulating how to submit an article by this contributionship.^2^ Finally, attaching the ACF generated from an automatic system at the head of academic papers would make it less possible for a snub by institutions (Fleming, 2021). A snub could potentially increase the recognition of authors’ credits more practically than their contribution statements.

## Author’s note

Contributionship has been pre-registered in Open Science Framework (OSF) at https://osf.io/3sjbc/ (10.17605/OSF.IO/3SJBC).

## Author contributions

ZC: conceptualization, data curation, formal analysis, funding acquisition, methodology, and writing—original draft. XRL: conceptualization and validation. KM, XYL, and XZ: methodology and validation. ZF: funding acquisition. HC-P: conceptualization and supervision. All authors contributed to the article and approved the submitted version.

## Footnotes

1
https://osf.io/3sjbc/

2
http://neurospider.cn/stimulatingsystem.html
